# Arrest Functions of the MIF Ligand/Receptor Axes in Atherogenesis

**DOI:** 10.3389/fimmu.2013.00115

**Published:** 2013-05-16

**Authors:** Sabine Tillmann, Jürgen Bernhagen, Heidi Noels

**Affiliations:** ^1^Institute of Biochemistry and Molecular Cell Biology, RWTH Aachen UniversityAachen, Germany; ^2^August-Lenz-Stiftung, Institute for Cardiovascular Research, Ludwig-Maximilians-University MunichMunich, Germany; ^3^Institute of Molecular Cardiovascular Research, RWTH Aachen UniversityAachen, Germany

**Keywords:** chemokine, leukocyte recruitment, arrest, signal transduction, atherosclerosis, inflammation

## Abstract

Macrophage migration inhibitory factor (MIF) has been defined as an important chemokine-like function (CLF) chemokine with an essential role in monocyte recruitment and arrest. Adhesion of monocytes to the vessel wall and their transendothelial migration are critical in atherogenesis and many other inflammatory diseases. Chemokines carefully control all steps of the monocyte recruitment process. Those chemokines specialized in controlling arrest are typically immobilized on the endothelial surface, mediating the arrest of rolling monocytes by chemokine receptor-triggered pathways. The chemokine receptor CXCR2 functions as an important arrest receptor on monocytes. An arrest function has been revealed for the *bona fide* CXCR2 ligands CXCL1 and CXCL8, but genetic studies also suggested that additional arrest chemokines are likely to be involved in atherogenic leukocyte recruitment. While CXCR2 is known to interact with numerous CXC chemokine ligands, the CLF chemokine MIF, which structurally does not belong to the CXC chemokine sub-family, was surprisingly identified as a non-cognate ligand of CXCR2, responsible for critical arrest functions during the atherogenic process. MIF was originally identified as macrophage migration inhibitory factor (this function being eponymous), but is now known as a potent inflammatory cytokine with CLFs including chemotaxis and leukocyte arrest. This review will cover the mechanisms underlying these functions, including MIF’s effects on LFA1 integrin activity and signal transduction, and will discuss the structural similarities between MIF and the *bona fide* CXCR2 ligand CXCL8 while emphasizing the structural differences. As MIF also interacts with CXCR4, a chemokine receptor implicated in CXCL12-elicited lymphocyte arrest, the arrest potential of the MIF/CXCR4 axis will also be scrutinized as well as the recently identified role of pericyte MIF in attracting leukocytes exiting through venules as part of the pericyte “motility instruction program.”

## Introduction

Leukocyte recruitment and arrest are central steps in inflammatory reactions and associated diseases, including atherosclerosis (Box 1). Identifying the main players mediating chemotaxis and arrest is therefore crucial. Boisvert et al. (1998) revealed an important role for the chemokine receptor CXCR2 in mediating monocyte recruitment into atherosclerotic lesions by showing a reduced lesion size and macrophage content in atherosclerosis-prone *Ldlr*^−/−^ mice transplanted with *Cxcr2*-deficient bone marrow. Although mice do not express an ortholog of the CXCL8/IL8 ligand of human CXCR2, the Cxcr2 ligand Cxcl1 (also known as KC/Gro-α) was detected in advanced lesions in mice (Boisvert et al., 1998)^1^. However, a subsequent study in 2006 showed that the reduction in lesion size in *Cxcl1*-deficient *Ldlr*^−/−^ mice didn’t exceed half of what was observed in the bone marrow-specific *Cxcr2* knock-out (Boisvert et al., 2006), suggesting the presence of other relevant Cxcr2 ligands with an important role in monocyte recruitment during atherogenesis. In fact, one such factor was uncovered a year later and was found to be the inflammatory cytokine macrophage migration inhibitory factor (MIF). MIF was originally discovered half a century ago as a T-cell-derived factor inhibiting the random migration of macrophages out of capillary tubes and thus was termed macrophage migration inhibitory factor. However, following its cloning and the biochemical characterization and preparation of MIF protein, MIF was later on redefined to be a pleiotropic inflammatory cytokine with critical roles in physiological immunity but also inflammatory diseases and cancer (Bernhagen et al., 1993; Calandra and Roger, 2003). Although the migration inhibitory activity of MIF was not studied and characterized much further, the eponymous name “MIF” was kept up over the years. It was thus largely unexpected, when MIF was identified as a ligand of CXCR2, exhibiting chemokine-like properties, and shown to be a crucial pro-atherogenic factor (Bernhagen et al., 2007). Intriguingly, MIF/CXCR2 interaction was found to trigger the recruitment and arrest of monocytes, whereas MIF-mediated T-cell recruitment could be traced to an interaction of MIF and yet another chemokine receptor. This was CXCR4, a CXC chemokine receptor thought to be much more specific regarding its ligand spectrum than CXCR2 (Bernhagen et al., 2007). Interestingly, MIF-mediated monocyte recruitment had previously been described in other inflammatory diseases, such as arthritis and glomerulonephritis (Lan et al., 1997; Morand et al., 2006), but was thought to represent an indirect event at the time. And although the third MIF receptor CD74 was already identified as a MIF-interacting membrane protein in 2003, a direct role of CD74 in MIF-mediated monocyte chemotaxis and arrest was not revealed until the discovery of CXCR2/CD74 complexes in 2007. Interestingly, it is now also clear that CD74 has a role in atherogenesis (Sun et al., 2010).

Box 1 **The leukocyte adhesion cascade.**

Leukocyte arrest on inflamed endothelium can be divided into three main steps: rolling, adhesion, and transmigration. Leukocyte rolling is mediated by the binding of leukocyte-derived PSGL1 to the selectins P-selectin and E-selectin on inflamed endothelial cells (ECs). Next, chemokines triggering their respective G protein-coupled receptor (GPCR) on the leukocyte cell surface promote leukocyte integrin activation, resulting in leukocyte arrest. Finally, leukocytes transmigrate across the endothelium into the vessel wall, which can occur by paracellular (through endothelial junctions) or transcellular route (through the EC body). This three-step model has been refined over the last years, to include tethering (capture), rolling, slow rolling, arrest, adhesion strengthening, intraluminal crawling, and transmigration (Ley et al., 2007).

This review discusses MIF’s role as a chemokine-like mediator, addressing its structure, receptor binding capacity and importance in leukocyte recruitment, particularly arrest, in the context of atherosclerosis.

## MIF as an Important Chemokine-Like Function – Chemokine

Chemokines are 8–12 kDa cytokines with chemotactic properties, playing a fundamental role in leukocyte trafficking (Bajetto et al., 2002; Weber et al., 2004; Charo and Ransohoff, 2006). Typically, a chemokine consists of a disordered N-terminus containing a characteristic cysteine motif, an N-loop region, three antiparallel β-strands linked by turns designated 30s-, 40s-, and 50s-loop, and a C-terminal α-helix, which together form the typical chemokine fold (Clark-Lewis et al., 1995). Chemokines share 20–50% gene and amino acid homology, and are classified into four groups depending on the presence and spacing of their N-terminal cysteine residues. These groups comprise the C, CC, CXC, and CXXXC chemokines, with the CXC and CC groups being most prominent (Murphy et al., 2000). The N-terminal cysteine motif stabilizes the chemokine structure by forming two disulfide bonds, one between the first cysteine with a cysteine in the 30s-loop, and the other one between the second cysteine and a cysteine in the 50s-loop (Fernandez and Lolis, 2002). Chemokines exert their specific function by binding to rhodopsin-like G protein-coupled receptors (GPCRs), which contain a seven-transmembrane domain and signal through heterotrimeric G proteins (Thelen and Didichenko, 1997; Murphy et al., 2000; Thelen, 2001; Bajetto et al., 2002; Charo and Ransohoff, 2006). The receptors are classified according to the chemokines they bind (Murphy et al., 2000). It has been suggested that for all chemokine sub-groups, the binding mechanism follows a so-called two-site-binding mechanism. First, there is an interaction between the N-loop of the chemokine with the N-terminus of the receptor (site I interaction). This results in a conformational change of the receptor and allows a second interaction between the N-terminus of the chemokine and the extracellular loops of the receptor (site II interaction) (Clark-Lewis et al., 1995; Rajagopalan and Rajarathnam, 2006).

In the last decade, there was a raising need to establish an additional chemokine category, to accomodate proteins that exhibit similar functions as the prototypical, “classical” chemokines, but that lack the typical chemokine structure. Characteristics of this group of “chemokine-like function (CLF) – chemokines” were defined as follows: (i) CLF chemokines are released during infection, inflammation, or cell stress by non-classical export or due to cell death; (ii) they do not usually share the typical chemokine fold and the N-terminal residues with the classical chemokines; (iii) they exhibit chemokine-like activities in particular promoting chemotaxis; and (iv) they typically interact with a GPCR, preferentially functioning as non-cognate ligand of a classical chemokine receptor (Degryse and de Virgilio, 2003; Yang et al., 2004; Oppenheim and Yang, 2005; Noels et al., 2009). Some representatives of this sub-group and their characteristic features are listed in Table 1.

**Table 1:** **Chemokine-like function (CLF) chemokines**.

Name	Secretion mechanism	Chemotaxis	Additional CLF feature	Interacting chemokine receptor	Other receptor	Reference
Aminoacyl-tRNA synthetases (AaRS), mini-tyrosyl-tRNA synthetase (mini-TyrRS)	Apoptosis/cell death	Monocytes, neutrophils, T-cells, immature DCs	ELR motif	CCR5, CCR3, CXCR1	–	Wakasugi and Schimmel (1999), Wakasugi et al. (2002), Yang et al. (2002)
Complement factor 5a (C5a)	–	DCs, monocytes, macrophages, neutrophils, eosinophils	Modulation of cytokine release	–	C5aR, C5L2	Wennogle et al. (1994), Sozzani et al. (1995), Riedemann et al. (2004), Gao et al. (2005)
Cyclophilin	Secretory pathway unknown (possibly non-classical)	Murine bone marrow cells, eosinophils, neutrophils, T-cells	Integrin-mediated adhesion of T-cells	–	CD147	Colley et al. (1991), Xu et al. (1992), Price et al. (1994), Allain et al. (2002), Yurchenko et al. (2002), Suzuki et al. (2006), Khromykh et al. (2007)
α-Defensins	Cell death	Immature DCs, memory, and CD8 T-cells	–	Chemokine receptor of unknown identity	–	Yang et al. (2001)
β-Defensins	Cell death	Immature DCs, memory and CD8 T-cells, monocytes	Augment cytokine production	CCR6	TLR4	Yang et al. (1999), Biragyn et al. (2002), Hoover et al. (2002), Oppenheim et al. (2003)
Cathelicidins (LL37, Cramp-1)	Cell death and possibly specific secretion	Monocytes, neutrophils, mast cells, T-cells	various	–	–	Yang et al. (2001), Soehnlein et al. (2011), Wantha et al. (2013)
High-mobility group binding protein-1 (HMGB-1)	Non-classical export/cell death	DCs, immature DCs, neutrophils, macrophages	Cytokine expression, modulation of VCAM1/ICAM1 expression	CXCR4 (in complex with CXCL12)	RAGE, TLR2/4	Andersson et al. (2000), Fiuza et al. (2003), Pullerits et al. (2003), Yang et al. (2007), van Zoelen et al. (2009), Schiraldi et al. (2012)
Macrophage migration inhibitory factor (MIF)	Non-classical export/(cell death?)	Monocytes, T-cells, neutrophils, EPCs, tumor cells	Pseudo-ELR motif	CXCR4, CXCR2 (CXCR7?)	CD74	Bernhagen et al. (2007), Noels et al. (2009), Cho et al. (2010), Dessein et al. (2010), Tarnowski et al. (2010)
Thioredoxin (TRX)	Non-classical export/apoptosis	Monocytes, neutrophils, T-cells	Cytokine expression	Unknown	TNF-R-super-family member 8 (TNFRSF8/CD30)	Bertini et al. (1999), Schwertassek et al. (2007)
Urokinase (uPa)	Non-classical export/secretory vesicles	Monocytes, keratinocytes, fibroblasts	–	–	FPRL1, uPAR	Quax et al. (1994), Takahashi et al. (1998), Resnati et al. (2002), Roychoudhury et al. (2006)
Y-box protein-1 (YB-1)	Non-classical export/apoptosis	Mesangial cells	–	–	Notch-3	Frye et al. (2009), Rauen et al. (2009)

*DC, dendritic cell; EPC, endothelial progenitor cell; FPRL1, formyl peptide receptor-like 1*.

MIF is a typical CLF chemokine, as missing cysteines in its N-terminus do not allow for a classification of MIF into one of the four prototypical chemokine classes, although MIF shares several features with chemokines (Box 2). As such, MIF mediates the recruitment of monocytes, T-cells, neutrophils, endothelial progenitor cells, and tumor cells (Ren et al., 2003; Gregory et al., 2006; Bernhagen et al., 2007; Takahashi et al., 2009; Brandau et al., 2010; Dessein et al., 2010; Simons et al., 2011). Furthermore, MIF is immobilized on the endothelial cell (EC) surface (Schober et al., 2004; Bernhagen et al., 2007), where it induces leukocyte arrest (Schober et al., 2004; Amin et al., 2006). Interestingly, MIF was shown to directly mediate these chemokine-like functions by triggering the activation of leukocytic integrins (Box 3) through the CXC chemokine receptors CXCR2 and CXCR4 on monocytes/neutrophils and T-cells, respectively (Bernhagen et al., 2007; Zernecke et al., 2008; Kraemer et al., 2012), as discussed in more detail below.

Box 2 **Macrophage migration inhibitory factor (MIF).**

Macrophage migration inhibitory factor (MIF) is a pleiotropic inflammatory cytokine with chemokine-like functions, thus placing it into the CLF chemokine class. The sequence of MIF differs by only one residue from a protein mediator called glycosylation-inhibiting factor (GIF). The name “macrophage migration inhibitory factor” goes back to the initial discovery of MIF in 1966, when MIF-containing T-cell supernatants were found to inhibit the random migration of guinea pig macrophages out of capillary tubes (David, 1966). As a CLF chemokine, MIF acts as a chemoattractant for leukocytes, endothelial progenitor cells, and certain tumor cells, and mediates many pro-inflammatory processes through the induction of cytokines, chemokines, and adhesion molecules. Also, MIF counteracts the anti-inflammatory activity of glucocorticoids. MIF has been implicated in a variety of acute and chronic inflammatory diseases like sepsis, atherosclerosis, rheumatoid arthritis, inflammatory lung disease, or systemic lupus erythematosus. MIF also is a tumor promoter in most models (Calandra and Roger, 2003). MIF signals through its high affinity receptor CD74, a surface form of the MHC class II invariant chain Ii, and through the chemokine receptors CXCR2 and CXCR4. Apart from CLF functions, MIF exhibits evolutionarily conserved catalytic activities as an oxidoreductase and tautomerase activity. The physiological and pathophysiological relevance of these catalytic functions, which can readily be detected *in vitro*, is still unclear (Kraemer et al., 2012). The tautomerase activity of MIF is shared by D-dopachrome tautomerase (D-DT), a protein with 34% amino acid homology to MIF in humans, and 27% in mice. D-DT also binds to CD74 and has a broad overlapping spectrum of functions and therefore was recently designated as MIF-2 (Merk et al., 2012). Unfortunately, MIF is frequently mixed up with another chemokine with a similar name, i.e., macrophage inflammatory protein-1α (MIP-1α or CCL3). This chemokine also acts in a pro-inflammatory manner and is produced and secreted by macrophages, but unlike MIF, MIP-1α is classified into the CC chemokine class and is formally not related to MIF by structure.

Box 3 **Integrins.**

Integrins are αβ heterodimeric transmembrane proteins mediating the arrest of cells to the extracellular matrix (ECM) or other cells through interaction with ECM proteins (e.g., fibronectin, laminin, collagen, and vitronectin) or integrin ligands (e.g., VCAM1, ICAM1), respectively. Currently, 19α and 8β subunits have been identified in vertebrates, which can be assembled into 24 different integrins. Well-studied are the β_1_-integrin VLA4 (α_4_β_1_) and the β_2_-integrins LFA1 (α_L_β_2_, CD11a/CD18) and MAC1 (α_M_β_2_, CD11b/CD18), which bind to VCAM1 and ICAM1, respectively (Zhang and Wang, 2012). The association strength between a specific integrin heterodimer and a ligand is called “integrin affinity.” It is dependent on the composition and conformation of the integrin, the latter being modulated by intracellular signaling events triggered by, e.g., GPCR stimulation. For LFA1, at least 3 different conformations have been demonstrated. In its “closed,” inactive state, the extracellular domain of LFA1 is bent towards the plasma membrane, with the ligand-binding headpiece situated close to the membrane, preventing ligand binding. An intermediate affinity of LFA1 has been linked to an “intermediate extended” state with a closed ligand-binding headpiece extending above the plasma membrane. The high affinity, “open” conformation of LFA1 is coupled to the “opening” of the ligand-binding headpiece through conformational rearrangements. This “open headpiece” of LFA1 has been shown to be necessary and sufficient for cell arrest under flow (Lefort and Ley, 2012).

Besides integrin affinity, cellular adhesion strength is affected by the integrin density, or valency, on the cell surface. This is regulated by integrin expression and clustering, and contributes to the joined, synergistic strength of all integrin-ligand interactions, called “integrin avidity” or “functional affinity.” In addition to intracellular signaling cascades regulating integrin affinity (called “inside-out signaling”), ligand binding by integrins also mediates “outside-in signaling” affecting cellular processes, as for example gene expression, cell proliferation and survival.

In addition, MIF indirectly enhances leukocyte arrest by inducing the expression of adhesion molecules or other chemokines. This has been observed for both endogenous and exogenous MIF on ECs and leukocytes, either by MIF stimulation alone, or by MIF in combination with other pro-inflammatory stimuli. For example, *Mif*-deficient mice show a reduced adhesion of leukocytes to the endothelium of the cremaster microvasculature upon injection of inflammatory agents such as Tnfα and lipopolysaccharide (LPS), or chemokines such as Cxcl1 or Ccl2 (Gregory et al., 2004; Fan et al., 2011; Santos et al., 2011). For TNFα, this could be linked to a reduced basal and TNFα-triggered expression of the integrin ligands VCAM1 and ICAM1, and of the cytokines IL6, CXCL8, and CCL2 in *MIF*-deficient ECs, possibly through a reduced TNFα-induced p38 activation in the absence of MIF (Cheng et al., 2010). Similarly, the cremaster muscle microvasculature of *Mif*-deficient mice showed a diminished LPS-induced Vcam1 expression pattern (Gregory et al., 2009). Also, Cxcl1-induced chemotaxis was significantly reduced in *Mif*-deficient neutrophils and was associated with a reduced mitogen-activated protein kinase (Mapk) activation (Santos et al., 2011). Similarly, Ccl2-triggered monocyte chemotaxis was severely decreased in the absence of endogenous Mif. This was associated with a reduced Rho GTPase and Mapk activation, and a reduced expression of the α4 integrin and of the Mapk-regulating protein Mkp1 in *Mif*-deficient macrophages (Fan et al., 2011). Likewise, exogenous MIF has been linked to an enhanced expression of chemokines and adhesion molecules. For example, injection of mice with recombinant MIF increased monocyte adhesion and endothelial transmigration in the microvasculature (Gregory et al., 2006; Fan et al., 2011). This was mostly dependent on Cd74 (Fan et al., 2011) and associated with enhanced Ccl2 secretion from microvascular ECs *in vitro*, without affecting endothelial Vcam1 expression *in vivo* (Gregory et al., 2006). In contrast, exogenous MIF upregulated ICAM1 on a human EC line *in vitro* (Lin et al., 2000) and reduced Icam1 expression was seen in the atherosclerotic aorta of mice treated with a Mif blocking antibody (Burger-Kentischer et al., 2006). These observations seem to be dependent on the vascular bed from which the ECs derive, as in contrast to the MIF-induced arrest responses on microvascular ECs, exogenous MIF could not induce leukocyte rolling or arrest on HUVECs. However, TNFα-induced leukocyte rolling and adhesion on HUVECs were enhanced by exogenous MIF, which could be linked with an increase in endothelial P-selectin expression, while the TNFα-induced expression of E-selectin, VCAM1, and ICAM1, or of different chemokines were unaltered in the presence of exogenous MIF (Cheng et al., 2010). Furthermore, MIF mediates neutrophil accumulation in MIF-triggered lung inflammation by inducing the chemokines Cxcl1 and Cxcl2/Mip2 in alveolar macrophages through Cd74/extracellular signal regulated kinase (Erk) signaling (Takahashi et al., 2009) and can promote neutrophil chemotaxis in particular in the presence of actively expressed surface CD74 (Bernhagen et al., 2007). In addition, MIF has been shown to increase the surface expression of ICAM1 and VCAM1 on human monocytes via PI3K/AKT, p38, and NF-κB (Amin et al., 2006), which can be shed to soluble adhesion molecules, capable of mediating leukocyte chemotaxis (Kitani et al., 1998; Tokuhira et al., 2000). In conclusion, MIF is a pivotal mediator of leukocyte chemotaxis and arrest, by both direct mechanism or through the induction of other chemokines or adhesion molecules.

In addition to its chemotactic and arrest properties, MIF exerts pro-inflammatory and anti-apoptotic functions, either through receptor activation by extracellular MIF (as described in more detail below) or through intracellular interactions, e.g., with JAB1/CSN5 or with the pro-apoptotic proteins BIM and p53 (Kleemann et al., 2000; Jung et al., 2008; Liu et al., 2008; Noels et al., 2009). Furthermore, comparable to other CLF chemokines, MIF is secreted upon diverse inflammatory or stress factors (Table 2). This secretion occurs through a non-classical pathway, a so-called export pathway, as MIF lacks a typical N-terminal consensus secretion sequence required for classical endoplasmic reticulum/Golgi-mediated protein secretion (Flieger et al., 2003). Of note, MIF secretion is not only observed in immune cells, but other cell types with a prominent role in atherogenesis including ECs and SMCs can also be triggered to secrete MIF (Bernhagen et al., 1994; Noels et al., 2012).

**Table 2:** **MIF expression and secretion in cell types relevant in atherogenesis**.

Cell type	Basal expression	MIF expression (secretion) upregulated by	Reference
Monocytes/macrophages	Yes	LPS, TNFα, IFNγ, CD40L, ATII, oxLDL, bacterial exotoxins, hypoxia, glucocorticoids	Calandra et al. (1994), Schmeisser et al. (2005), Burger-Kentischer et al. (2002), Calandra et al. (1998), Schmeisser et al. (2005), Calandra et al. (1995)
T-cells	Low	T-cell activation (αCD3, PMA/ionomycin) glucocorticoids	Bloom and Bennett (1966), Bacher et al. (1996), Bacher et al. (1996)
B-cells	Yes	Tumor stress signals	Wymann et al. (1999), Reinart et al. (2013)
ECs	Low	LPS, oxLDL, hypoxia, thrombin	Nishihira et al. (1998), Burger-Kentischer et al. (2002), Schober et al. (2004), Schmeisser et al. (2005), Simons et al. (2011), Zhang et al. (2012), Shimizu et al. (2004)
SMCs	Low	oxLDL, hypoxia	Chen et al. (2009), Zhang et al. (2012)

*LPS, lipopolysaccharide; TNF, tumor necrosis factor; IFN, interferon; ATII, angiotensin II; oxLDL, oxidized low density lipoprotein; ECs, endothelial cells; SMCs, smooth muscle cells*.

## Structure – Function Relationships of MIF as a CXCR2 Ligand

MIF is a conserved protein, ubiquitously expressed in mammals. Furthermore, MIF homologs have been identified in avians, fish, plants (*Arabidopsis thaliana*), the nematode *Caenorhabditis elegans*, cyanobacteria, ticks, and parasites, amongst others (Calandra and Roger, 2003; Kim et al., 2010). The human and mouse MIF protein consist of 114 amino acids (excluding an N-terminal methionine, which is processed after ribosomal synthesis), with a total molecular weight of 12.3 kDa. The human and mouse orthologs share a 90% sequence homology and lack a conventional leader sequence targeting proteins for classical secretion (Bernhagen et al., 1994). In addition to cytokine and chemokine-like activities, MIF is unusual in featuring two evolutionarily conserved catalytic activities, being an oxidoreductase and a tautomerase activity. Despite its consensus Cys-Xaa-Xaa-Cys motif, structural similarities between MIF and oxidoreductases such as thioredoxin or glutaredoxin are of remote nature only. In contrast, MIF possesses three-dimensional structural homology with the bacterial isomerases 4-oxalocrotonate-tautomerase, 5-carboxymethyl-2-hydroxymuconate isomerase, and chorismate mutase (Rosengren et al., 1997; Kleemann et al., 1998; Calandra and Roger, 2003). Furthermore, MIF’s overall architecture resembles that of human D-dopachrome tautomerase (D-DT). Also, MIF and D-DT share overlapping biological functions, both bind the CD74 receptor, and activate similar signaling pathways (Sugimoto et al., 1999; Merk et al., 2012). Therefore, “MIF-2” was recently suggested as an alternative name for D-DT (Merk et al., 2012). Remarkably, a MIF ortholog can even be found in the hookworm *Ancylostoma ceylanicum* (aceMIF), sharing 28–35% sequence homology with human MIF. Like the human ortholog, aceMIF shows tautomerase activity, binds to CD74 and has chemoattractant properties (Cho et al., 2010). Although the global topology is similar to human MIF, protein surface and electrostatic potential are distinct (Cho et al., 2007).

**Figure 1:**
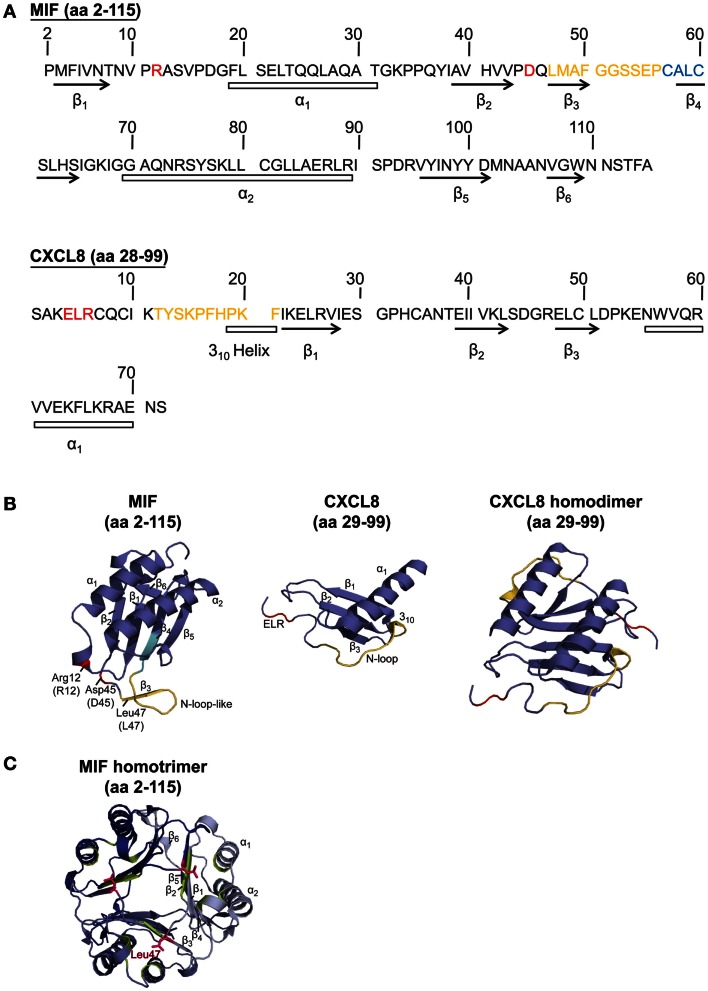
**Sequence and structure comparison of human MIF and the cognate CXCR2 ligand CXCL8**. **(A)** Amino acid (aa) sequence comparison of MIF and CXCL8. **(B,C)** Comparison of the crystal structure of the MIF monomer, CXCL8 monomer, CXCL8 dimer, and MIF trimer. To **(A,B)**: CXCL8 (aa 28–99) is the predominant form of CXCL8. α-Helices and β-sheets are indicated. Important amino acids and motifs are highlighted: ELR or pseudo-(E)LR (red); N-loop (for CXCL8) or N-like loop (for MIF; orange); CALC motif, forming the catalytic center of MIF’s oxidoreductase activity (blue). The MIF structural information is according to Orita et al. (2001), the crystal structure for CXCL8 was based on data from Clore et al. (1990). **(C)** Crystal structure of the MIF homotrimer (Orita et al., 2001), showing the barrel-shaped homotrimeric structure and the inter-subunit interactions between two β-strands of one subunit with β-sheets of adjacent subunits. Further stabilization is provided by the hydrophobic interaction of Leu47 (pink) of the β3-strand of one subunit with an adjacent hydrophobic pocket (green) on a second subunit, comprising amino acids mainly positioned on the β2-strand. For details, see text.

The MIF monomer consists of two antiparallel α-helices and a four-stranded β-sheet (excluding two really short β-strands), as displayed in Figure 1. Although MIF has no sequence homology to other chemokines, the 3D structure of the MIF monomer resembles the dimeric form of CXCL8 and other CXC chemokines (Weber et al., 2008) (Figure 1). But, the monomer is not the only existing form of the MIF protein. Nuclear magnetic resonance (NMR) analysis suggested that MIF dimerizes, whereas X-ray crystallography revealed human MIF as a trimer. The barrel-shaped homotrimeric structure is stabilized by conserved inter-subunit interactions between two β-strands of one subunit with β-sheets of adjacent subunits (Sugimoto et al., 1996; Sun et al., 1996a). Further stabilization is provided by the hydrophobic interaction of Leu47 on the β3-strand of one subunit with an adjacent hydrophobic pocket on a second subunit, comprising amino acids mainly positioned on the β2-strand (El-Turk et al., 2012) (Figure 1). However, X-ray and NMR structural analyses are performed at mg/ml concentrations of the analyte, which are far off the physiological concentrations found in the cell or in extracellular fluids. Overall, the physiological oligomerization state of MIF remains elusive. Crosslinking studies revealed the coexistence of MIF monomers, dimers, and trimers (Sugimoto et al., 1996; Sun et al., 1996a,b), probably influenced by local MIF concentrations. At physiological conditions, an equilibrium of monomers and dimers has been described, whereas at concentrations >10 μg/ml, trimeric or higher-ordered oligomers seem to be preferred (Mischke et al., 1998; Calandra and Roger, 2003; Philo et al., 2004; El-Turk et al., 2008). Interestingly, homotrimeric MIF seems to drive inflammatory responses in the corneal epithelium, as recently reported by Reidy et al. (2013). Nevertheless, it is unlikely that the homotrimer is the only active form, as disruption of the homotrimeric structure with the MIF inhibitor ebselen leads to an increased chemotactic response (Ouertatani-Sakouhi et al., 2010). In addition, concentration extrapolations into the physiological ng/ml range would likely favor a predominant population of the monomeric state. Yet, monomeric MIF is thought to be intrinsically unstable, necessitating yet unknown mechanisms for its stabilization (Bernhagen and Lue, unpublished observations).

To study the structure-activity relationship of MIF in the context of its interaction with CXCR2, it appeared obvious to look for homologous structural features with the cognate CXCR2 ligand CXCL8, which, in its dimeric form, shares structural homology with the MIF monomer (Figure 1). Bioinformatic prediction analysis in conjunction with mutational studies revealed an important receptor interacting motif in MIF that resembles the N-terminal Glu-Leu-Arg (ELR) motif carried by a sub-group of CXC chemokines, as shown for CXCL8. This MIF motif, consisting of Asp45-X-Arg12, was termed “pseudo-(E)LR” motif, as the glutamate (Glu/E) was substituted with an aspartic acid (Asp/D) (Hebert et al., 1991; Weber et al., 2008). The non-adjacent residues of this pseudo-(E)LR motif are located in neighboring loops of the MIF protein with similar spacing as in the true ELR motif (Figure 1). Site-directed mutagenesis studies showed an almost complete inhibition of CXCR2 binding when the R12A or D45A mutation was introduced into MIF. The MIF-R12A mutant also exhibited a complete loss of chemotactic and arrest function. Also, the MIF-D45A mutant showed a reduced chemotactic and arrest activity in *in vitro* assays, whereas its hyperactivity towards neutrophil recruitment in a peritonitis model was shown to be CXCR4-mediated (Weber et al., 2008). Furthermore, evidence became available for an interaction of the pseudo-(E)LR motif with the extracellular loops EL-2 and EL-3 of CXCR2 (Kraemer et al., 2011). Still, it should be noted that Arg12 and Asp45 of the pseudo-(E)LR motif are located close to the critical Leu47 residue, which is involved in inter-subunit hydrophobic interactions modulating the conformation and stability, but not the oligomerization state, of homotrimeric MIF (El-Turk et al., 2012) (Figure 1). The reduced leukocyte adhesion activity, which was observed for the pseudo-(E)LR mutants (Weber et al., 2008), might thus not solely result from changes in the direct MIF receptor interaction locus, but could also result in part from modifications of the conformational stability of MIF by disturbance of the Leu47 region.

Interestingly, Kraemer et al. (2011) revealed the involvement of an N-like loop in MIF in binding to CXCR2. This loop spans 10 amino acids from position 47 to 56 but is structurally different from the N-loop of CXC chemokines (Figure 1; Table 3). Whereas the classical N-loop found in CXC chemokines contains 1–3 basic residues and interacts with the N-terminus of the receptor, the N-like loop of MIF has an acidic isoelectric point (pI) and interacts with EL-1 and parts of EL-2 as well as with the N-terminus of CXCR2, according to peptide spot array analysis. Importantly, short MIF N-like loop-derived peptides blocked monocyte arrest and inhibited MIF/CXCR2 interaction in a receptor competition assay, verifying the importance of the N-like loop of MIF for CXCR2 binding (Kraemer et al., 2011). Furthermore, amino acids in the region between residues 50 and 68 are critical for obtaining potent MIF neutralizing antibodies, which block important biological activities such as cell proliferation and glucocorticoid overriding *in vitro* and MIF-driven septic responses *in vivo* (Kerschbaumer et al., 2012). This confirmed that the N-like loop region of MIF is critical for MIF-driven receptor-mediated processes. Moreover, site-specific mutations of the cysteines at positions 57 and 60 and the use of peptides covering the region 50–65 further underscored that the sequence region of the N-like loop, i.e., region 47–68, is critical for a variety of MIF activities (Kleemann et al., 1998, 1999; Nguyen et al., 2003).

**Table 3:** **The N-like loop of MIF shows only limited similarity with the N-loop of CXC chemokines**.

Chemokine	N-loop sequence
CXCL1	LQTLQ GIHP
CXCL2	LQTLQ GIHL
CXCL3	LQTLQ GIHL
CXCL5	LQTTQ GVHP
CXCL6	LRVTL RVNP
CXCL7	IKTTS GIHP
CXCL8	IKTYSKPFHP
MIF	LMAFGGSSEP

*Adapted from Kraemer et al. (2011)*.

Taken together, as suggested by Kraemer et al. (2011), the binding of MIF to CXCR2 seems to follow a two-site-binding mechanism which is similar but not identical to that between CXCL8 and CXCR2. In contrast, no structure-activity relationship data are available yet for the interaction between MIF and its receptors CD74 and CXCR4. Interesting questions are therefore, whether the uncovered motifs mediating the MIF/CXCR2 interaction are also important for the interaction between MIF and CXCR4, whether for CD74 binding fully different regions are required, and whether CXCR7, for which an interaction with MIF has recently been implied, binds directly to MIF and utilizes the N-like loop and pseudo-(E)LR motif as well.

Interestingly, the anti-inflammatory drug AV411 (Ibudilast) and its analog AV1013, both allosteric inhibitors of MIF’s tautomerase activity, were found to inhibit MIF-mediated CXCR2-dependent chemotaxis of monocytes (Cho et al., 2010). AV1013 binds into a pocket formed by several C-terminal residues of MIF. AV1013 binding apparently induces conformational changes leading to both an inactivation of the tautomerase site and changes at the MIF/CXCR2 interface, i.e., likely affecting the N-like loop or pseudo-(E)LR motif. Alternatively, conformational changes in the tautomerase site could subsequently lead to conformational changes in the receptor interaction interface of MIF (Cho et al., 2010). Similarly, the hormonally inert isomer DT(4) of the thyroid hormone thyroxine [T(4)], inhibited MIF’s tautomerase activity by binding to a hydrophobic pocket harboring this enzymatic function and reduced leukocyte accumulation in a carrageenan-induced airpouch model in wildtype but not *Mif*^−/−^ mice, providing further evidence for a potential participation of the tautomerase site in MIF receptor-mediated chemotaxis (Al-Abed et al., 2011). Yet, disruption of the MIF trimer, and therefore of the active tautomerase site^2^, increased MIF-mediated chemotaxis (Ouertatani-Sakouhi et al., 2010). In addition, Fingerle-Rowson et al. (2009) showed that the tautomerase-inactive mutant P2G-MIF, which contains a mutation of the crucial catalytic N-terminal Proline (Pro2)^3^, could still bind CD74 and mediate growth regulation in a skin tumorigenesis model, although to a somewhat reduced level. This indicates that the enzyme activity *per se* is not essential for CD74 receptor binding. Also, in comparison with wildtype MIF, the capacity of P2G-MIF to compete with CXCL8 for binding to CXCR2-expressing cells was more reduced than ligand competition in the CD74 binding assay (Fingerle-Rowson et al., 2009). This suggests that the Pro2 residue and/or conformational changes in the tautomerase site affect MIF binding to CXCR2 more than the MIF-CD74 interaction.

## MIF’s Arrest Function through Its Receptor CXCR2

MIF, immobilized on the endothelial surface, triggers the arrest of monocytes/neutrophils and T-cells through CXCR2 and CXCR4, respectively, by a rapid and transient activation of the leukocyte integrins LFA1 and VLA4 (Box 3) (Bernhagen et al., 2007). While the precise mechanism of MIF deposition on ECs has not yet been explored, the basic pI could be a likely explanation. Alternatively, ECs express CD74 which has been found to be modified by chondroitin sulfate, thus providing a possible anchoring site for MIF as well.

**Figure 2:**
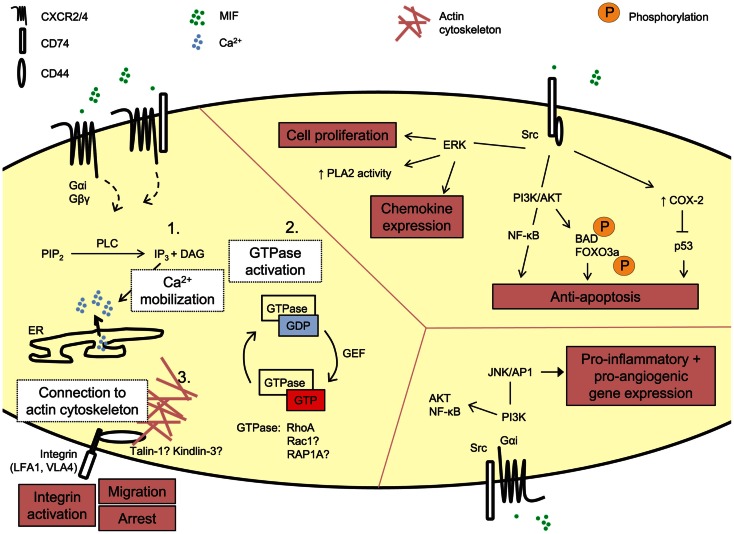
**Signaling by exogenous MIF**. MIF can induce signaling cascades through its receptors CD74, CXCR2, and CXCR4. These pathways underlie MIF’s biological functions, e.g., leukocytic integrin activation, cell proliferation, and anti-apoptosis, induction of pro-inflammatory gene expression. The detailed molecular mechanism underlying MIF’s arrest function through its receptors CXCR2 and CXCR4 is still unexplored. Three main steps in GPCR-mediated integrin activation can be distinguished, i.e., PLC-mediated calcium mobilization, activation of small GTPases and recruitment of actin-binding proteins linking the integrin to the actin cytoskeleton. PIP2, phosphatidylinosytol 4,5-biphosphate; PLC, phospholipase C; IP3, inosytol 1,4,5-triphosphate; DAG, diacylglycerol; Ca^2+^, calcium; ER, endoplasmic reticulum; GDP, guanosine diphosphate; GTP, guanosine triphosphate; guanine nucleotide exchange factor; PLA2, phospholipase A2; ERK, extracellular signal-related kinase; PI3K, phosphatidylinositol 3-kinase; NF-κB, nuclear factor-κB; BAD, BCL2-associated agonist of cell death; FOXO3A, forkhead box O3a; COX-2, cytochrome C oxidase subunit 2; JNK, c-Jun N-terminal kinase; AP1 (c-Jun), activator protein-1.

For immobilized classical chemokines, the GPCR/Gαi-mediated intracellular signaling cascade in leukocytes triggering integrin activation and leukocytic arrest, has been shown to be very complex, with currently 65 proteins identified to be possibly involved (Ley et al., 2007; Montresor et al., 2012). Three main stages of integrin activation are distinguished. These involve: (1) phospholipase C (PLC)-mediated calcium influx, (2) small GTPases, and (3) actin-binding proteins as talin-1 and kindlin-3, as described in more detail elsewhere (Ley et al., 2007; Lefort and Ley, 2012; Montresor et al., 2012) (Figure 2). However, this model of GPCR-mediated integrin activation cannot be universally applied to all conditions, and it is expected to be dependent on the GPCR, GPCR ligand, the integrin activated, and the biological context (Ley et al., 2007; Montresor et al., 2012). For MIF/CXCR2, few details are known about the exact molecular delineation of proteins involved in MIF receptor-mediated integrin activation. Initial inhibitor studies revealed MIF to mediate monocyte and T-cell adhesion by Gαi proteins and PI3K (Bernhagen et al., 2007), a kinase which is directly activated by the Gβγ dimer and which mediates adhesion stabilization of neutrophils to CXCL1 through CXCR2 (Smith et al., 2006). Also, MIF triggered CXCR2-dependent calcium transients. In mouse fibroblasts, recombinant MIF induces the activation of RhoA GTPase and Rho kinase, and *Mif*-deficient fibroblasts showed a reduction in RhoA GTPase activation and stress fiber formation (Swant et al., 2005). Of note, the latter has been linked with integrin clustering (Roovers and Assoian, 2003). Also VASP, LASP-1, IQGAP1, and NHERF1 were recently identified to interact with CXCR2 and to be involved in CXCL8-triggered chemotaxis (Neel et al., 2009, 2011; Wu et al., 2012), but it remains unknown whether this is directly linked to integrin activation, or whether these proteins are also involved in MIF/CXCR2-mediated arrest.

In addition to this direct link of MIF-CXCR2 signaling towards integrin activation, MIF has been shown to induce the migration of human chondrosarcoma cells by upregulating the transcription of the α_v_β_3_ integrin through PI3K/AKT/NF-κB signaling in a CXCR2- and CXCR4-mediated way (Lee et al., 2012). This again indicates MIF’s potential to mediate chemokine-like functions through indirect effects, by regulating the expression of proteins involved in leukocyte adhesion, as discussed previously.

## CD74 and CXCR4 as Alternative MIF Receptors

### CD74

In 2003, the CD74 protein was discovered as a high affinity receptor for MIF (Leng et al., 2003). CD74 is well-known as an MHC class II chaperone, as it is the membrane-expressed portion of the invariant chain (II), which typically regulates antigenic peptide loading to MHC class II proteins through its CLIP domain. However, CD74 can also be expressed in the absence of the MHC class II protein, thus executing functions as membrane receptor (Borghese and Clanchy, 2011). The CD74 receptor is a type II membrane-spanning protein with a short cytoplasmic N-terminus. As a result, accessory signaling molecules like Src, CD44, c-Met, or other co-receptors are necessary to mediate CD74 signaling by MIF, i.e., by forming a functional receptor-tyrosine-kinase-(RTK)-like complex (Bernhagen et al., 2007; Gordin et al., 2010). Signaling of MIF through CD74 has been linked with MIF’s pro-inflammatory and anti-apoptotic functions. For instance, interaction of MIF with CD74 leads to the activation of MAPKs and other protein kinases. One example is the sustained and transient activation of the MAPK ERK1 and ERK2 (Lue et al., 2006; Shi et al., 2006). Sustained ERK activation is mediated by CD74/CD44 and protein kinase A and has been linked to cell proliferation and enhanced pro-inflammatory phospholipase A2 activity (Mitchell et al., 1999; Lue et al., 2006; Shi et al., 2006). Recently, also β-arrestin-1 was shown to be involved in MIF-triggered sustained ERK activation, mediating MIF internalization in a CD74- and clathrin-dependent manner (Xie et al., 2011). Another example is the effect of MIF and CD74 on the MAPK Jun N-terminal kinase (JNK). MIF either impedes JNK signaling and JNK-mediated apoptosis, or rapidly initiates JNK activation through CXCR4/CD74, activating the Src/PI3K/JNK/AP1 pathway, which results in the expression of the pro-inflammatory protein CXCL8 (Kleemann et al., 2000; Qi et al., 2009; Lue et al., 2011). Also, MIF-CD74 signaling has been shown to promote B-cell survival through CD44/PI3K/AKT-mediated NF-κB activation and NF-κB-induced CXCL8 secretion (Binsky et al., 2007; Gore et al., 2008). Furthermore, a CD74/Src/PI3K/AKT pathway links MIF to the phosphorylation of the pro-apoptotic proteins BAD and FOXO3a, providing a survival signal (Lue et al., 2007). Also, cyclooxygenase (COX)-2, which prevents the accumulation of p53, can be upregulated through this pathway, likewise contributing to survival (Mitchell et al., 2002).

In addition, CD74 has been shown to be involved in MIF-mediated leukocyte chemotaxis and arrest, although not in all circumstances. The administration of a CD74 blocking antibody reduced the MIF-dependent monocyte arrest on *ex vivo* carotid arteries from atherosclerotic mice (Bernhagen et al., 2007), and activation of macrophage CD74 by MIF leads to an ERK1/2-dependent release of Cxcl1 and Cxcl2, mediating the MIF-induced accumulation of neutrophils in the alveolar space (Takahashi et al., 2009). Furthermore, Ccl2-triggered monocyte arrest and endothelial transmigration were severely decreased in *Mif*- and *Cd74*-deficient mice. *Mif*- and *Cd74*-deficient macrophages also showed a reduced Ccl2-triggered chemotaxis *in vitro*, which was associated with a reduced Rho GTPase and Mapk activation. However, *Mif*-deficient macrophages showed a lower expression of the α_4_ integrin and of the Mapk-regulating protein Mkp1, which was not the case for *Cd74*-deficient macrophages, suggesting that the involvement of endogenous Mif and Cd74 in Ccl2-induced monocyte recruitment or adhesion could at least partially be mediated through distinct mechanisms. Furthermore, exogenous MIF could restore deficient Ccl2-triggered leukocyte adhesion, but not endothelial transmigration, in *Mif*^−/−^ and *Cd74*^−/−^ mice to the same extent, indicating that exogenous MIF enhances CCL2-mediated leukocyte adhesion mostly independently of CD74. In contrast, exogenous MIF-induced leukocyte adhesion and transmigration were severely impaired in *Cd74*^−/−^ mice (Fan et al., 2011), suggesting an alternative mechanism underlying MIF- versus CCL2/MIF-induced leukocyte adhesion *in vivo*. Alternatively, these different observations could result from differentially targeting vascular versus leukocytic cells to a different degree, when examining MIF-induced versus CCL2/MIF-induced leukocyte adhesion, respectively. Of note, MIF also desensitized CCL2-mediated chemotaxis (Hermanowski-Vosatka et al., 1999), implying a MIF/CXCR-mediated cross-signaling mechanism as known for classical chemokines.

### CXCR4

Another chemokine receptor known to interact with MIF is CXCR4, as shown by Bernhagen et al. (2007). Prior to this finding, the CXCL12/CXCR4 interaction was thought to be highly specific, from the receptor as well as from the ligand site (Murphy et al., 2000). The MIF/CXCR4 interaction together with the identification of the CXCL11 receptor CXCR7 as an additional receptor for CXCL12 (Balabanian et al., 2005; Burns et al., 2006; Thelen and Thelen, 2008), disproved the existence of a non-promiscuous interaction. In fact, CXCR4 is also known to interact with the HIV gp120 protein and a recent report suggests that serum ubiquitin also interacts with CXCR4 (Saini et al., 2010). CXCR4 is widely expressed, particularly on many cell types of the immune system (Murphy et al., 2000) (Table 4). The knock-out of *Cxcr4* in mice is embryonically lethal, due to defects in hematopoiesis, vasculo-, cardio-, and neurogenesis (Ma et al., 1998; Zou et al., 1998). CXCR4 has prominently been implicated in cell recruitment processes, with CXCL12 mediating the recruitment of hematopoietic and vascular progenitor cells from the bone marrow (Mohle et al., 1998; Sainz and Sata, 2007). On the other hand, MIF has been shown to induce T-cell recruitment and arrest through CXCR4-induced, rapid α_4_β_1_ (VLA4) integrin activation (Bernhagen et al., 2007). MIF was also identified as the critical autocrine CXCR4 ligand driving cell invasion by drug-resistant colon carcinoma HT-29 cells (Vera et al., 2008; Dessein et al., 2010). Little is known about the molecular details of MIF/CXCR4 signaling. In T-cells, MIF stimulation increases CXCL8 expression through both CXCR4 and CD74, depending on Src, PI3K, and JNK phosphorylation (Lue et al., 2011). Earlier on, CXCR4/CD74 heterodimers were found in monocytes, T-cells and fibroblasts, and MIF-induced AKT signaling was shown to be reduced both by blocking CD74 and CXCR4, indicating a functional CXCR4/CD74 MIF receptor complex (Schwartz et al., 2009).

**Table 4:** **MIF receptor expression in cell types relevant in atherogenesis**.

Cell type	Receptor	Remark	Reference
Monocytes/macrophages	CXCR2		Murphy et al. (2000)
	CXCR4		Murphy et al. (2000), Sunderkotter et al. (2004), Ingersoll et al. (2010)
	CD74		Martin-Ventura et al. (2009)
Neutrophils	CXCR2		Murphy et al. (2000)
	*CXCR4*	Upon stimulation	Bruhl et al. (2003)
	No CD74		
T-cells	CXCR4		Murphy et al. (2000)
	*CXCR2*	On some CD8^+^ T-cells, not on CD4^+^ T-cells	Chuntharapai et al. (1994)
	*CD74*	On a subset of activated T-cells	Stein et al. (2007)
B-cells	No CXCR2		Chuntharapai et al. (1994)
	CXCR4		Nie et al. (2004)
	CD74		Gore et al. (2008)
ECs	CXCR2		Murdoch et al. (1999)
	CXCR4		Gupta et al. (1998)
	*CD74*	Only upregulated under inflammatory stimulation	Stein et al. (2007)
SMCs	CXCR2		Govindaraju et al. (2006)
	CXCR4		Schecter et al. (2001)
	*CD74*	In atherosclerotic plaques	Martin-Ventura et al. (2009)

*ECs, endothelial cells; SMCs, smooth muscle cells*.

### CXCR7

Tarnowski et al. (2010) implicated a recently identified chemokine decoy receptor in MIF internalization and MIF-dependent adhesion of rhabdomyosarcoma cells. This seven-transmembrane-receptor, encoded by the *RDC-1* gene, was named CXCR7 and characterized as a receptor for CXCL11 and CXCL12 (Balabanian et al., 2005; Burns et al., 2006). CXCR7 is expressed on a variety of cells, including leukocytes, activated ECs, mature neurons, CD34^+^ progenitor cells, and several tumor cell lines (Balabanian et al., 2005; Burns et al., 2006; Infantino et al., 2006; Zabel et al., 2009; Hattermann et al., 2010; Tarnowski et al., 2010; Shimizu et al., 2011). Unlike the prototypical chemokine receptors, CXCR7 carries two amino acid substitutions in the DRYLAIV motif (A/S and V/T) on the second intracellular loop, resulting in a change in the adaptor motif for G proteins and thus to a loss of G protein signaling (Zabel et al., 2009). Even though typical chemokine receptor signaling pathways, like calcium mobilization, are absent for CXCR7, other signaling cascades have been described. For example, the β-arrestin-dependent internalization of chemokines by CXCR7 results in the activation of MAPK signaling and the CXCL12/CXCR7 interaction promotes Gαi-independent ERK and AKT phosphorylation, mediating T-cell chemotaxis and survival (Balabanian et al., 2005; Rajagopal et al., 2010; Kumar et al., 2012), although these findings have been controversial in part. On the other hand, CXCL11 binding to CXCR7 inhibits the CXCL12/CXCR4-mediated transendothelial migration of breast cancer cells (Dambly-Chaudiere et al., 2007; Boldajipour et al., 2008; Zabel et al., 2009). Thus, CXCR7 might play a role in MIF-mediated chemotaxis by generating MIF gradients leading to differential signaling, act as a co-receptor or influence chemokine crosstalk. Whether CXCR7 is implicated in a direct interaction with MIF or whether the observed effects by Tarnowski et al. (2010) are based on an indirect interaction, for instance by forming a functional complex with CXCR4 as identified by Luker et al. (2009), will have to be elucidated in the future. Nevertheless, the interaction of MIF with CXCR7 might be a further fine-tuning mechanism in the complex chemokine/chemokine receptor system.

### Receptor oligomerization

Receptor oligomerization is a further possibility to modulate ligand affinity, ligand internalization and signal transduction with an impact on cellular processes like cell arrest or cell activation. It is well established that chemokine receptors form dimers or even higher-order oligomers (Milligan, 2007; Thelen et al., 2010; Kraemer et al., 2013). Also, the MIF receptors CXCR2 and CXCR4 have been shown to homodimerize and even multimerize in a ligand-independent manner (Trettel et al., 2003; Hamatake et al., 2009; Wu et al., 2010). Moreover, CXCR2 and CXCR4 heterodimerize with various CXC receptors. For example, the MIF receptors CXCR4 and CXCR7 form a complex, thus modulating CXCL12-mediated CXCR4-dependent chemotaxis (Thelen and Thelen, 2008; Levoye et al., 2009). Also, CXCR2 heterodimerizes with CXCR1 (Wilson et al., 2005). Heterocomplex formation is not solely restricted to receptors of the same sub-family, but also exists between different chemokine receptor subtypes (Kraemer et al., 2013). The cooperation of the CXCR4 and CCR5 receptor, for example, is required for chemokine-induced T-cell stimulation at the immunological synapse (Contento et al., 2008). Furthermore, complexes between chemokine receptors and other receptor types were observed. As such, CXCR4 engages with the dopamine receptor and for CXCR2, a complex with the δ-opiod receptor (DOP) was demonstrated (Parenty et al., 2008; Kraemer et al., 2013). Interestingly, the CD74 receptor interacts with both CXCR2 and CXCR4 (Bernhagen et al., 2007; Schwartz et al., 2009). CXCR2/CD74 heterodimers are implicated in leukocyte recruitment. In this context, it was suggested that CD74 amplifies MIF/CXCR2-mediated signaling, as neutrophils, which lack CD74, only show a weak migratory response to MIF, whereas HL-60 cells, which do not express detectable levels of CD74, increasingly migrate to MIF after ectopic CD74 expression (Bernhagen et al., 2007). CXCR4/CD74 heterodimers were found in monocytes, T-cells, and fibroblasts. Both CXCR4 and CD74 mediate MIF-induced AKT signaling and a fast and transient activation of the JNK/AP1 pathway, suggesting the existence of a functional heterocomplex (Schwartz et al., 2009; Lue et al., 2011). Taken together, MIF/CXCR interactions play a role in inflammation and inflammatory leukocyte recruitment and arrest. The possibility that MIF interacts not only with a single receptor, but with a complex of receptors could further add to a highly controlled cell-, site- and disease-stage specific inflammatory cell adhesion process.

## MIF in Atherosclerosis

Atherosclerosis is caused and sustained by inflammatory processes in the vessel wall. The deposition and oxidation of low density lipoprotein (LDL) in the intima drives EC and SMC activation, and the recruitment and infiltration of leukocytes (Weber and Noels, 2011). Whereas MIF is only detectable at low levels in healthy vessels, hyperlipidemia strongly enhances MIF expression in ECs, SMCs, monocytes, and T-cells in atherosclerotic lesions (Lin et al., 2000; Burger-Kentischer et al., 2002, 2006), and an even further upregulation during atheroprogression suggested a role for MIF in plaque destabilization (Burger-Kentischer et al., 2002) (Table 5). *In vitro*, leukocytes and vascular cells have been shown to express MIF upon several inflammatory triggers (Table 2). Typically, an initial secretion pulse of preformed MIF protein precedes *MIF* transcription (Simons et al., 2011). Also, all cell types involved in atherogenesis, including monocytes/macrophages, neutrophils, B- and T-lymphocytes, ECs, and SMCs, express at least one of the MIF receptors CD74, CXCR2, or CXCR4 (Table 4), suggesting that they do not solely act as MIF storage pools, but also respond to secreted MIF.

**Table 5:** **MIF in atherosclerosis**.

			Reference
**MIF EXPRESSION IN ATHEROSCLEROSIS AND RESTENOSIS**			
*Native or diet-induced atherosclerosis*			
Rabbit	Upregulated in macrohages, ECs, and SMCs from early atherosclerotic lesions		Lin et al. (2000)
*Apoe*^−/−^ mouse	Enhanced in all cell types (monocytes, T-cells, ECs, SMCs), but mostly in monocytes		Burger-Kentischer et al. (2006)
Human	Enhanced in all cell types (monocytes, T-cells, ECs, SMCs)		Burger-Kentischer et al. (2002)
	Further upregulated upon progression	
*Injury-induced restenosis*			
*Apoe*^−/−*-*^; *Ldlr^−/−^*	Upregulated in medial SMCs (early) and ECs and foam cells (late)		Chen et al. (2004), Schober et al. (2004)
**EFFECTS OF MIF BLOCKADE ON ATHEROSCLEROSIS IN MICE**			
*Native or diet-induced atherosclerosis*			
*Mif*^−/−^; *Ldlr*^−/−^	High-fat diet	Smaller and less progressed lesions	Pan et al. (2004)
		Reduced cell proliferation	
		Reduced cathepsin expression	
*Mif*^−/−^; *Ldlr*^−/−^	Chow diet	Reduced lesion size	Verschuren et al. (2009)
		Reduced macrophage content	
*Apoe*^−/−^ +Mif blocking Ab	Chow diet	Only non-significant reduction in aortic lesion size	Burger-Kentischer et al. (2006)
		Reduced macrophage content	
		Reduced aortic expression of pro-inflammatory markers (CD40L, TNFα, IL12, ICAM1), the transcription regulators C-EBPβ and phospho-cJun, and of MMP2	
Atherosclerotic *Apoe*^−/−^ +Mif blocking Ab	High-fat diet	Regression in established lesions	Bernhagen et al. (2007)
		Reduced macrophage and T-cell content	
*Injury-induced restenosis*			
*Ldlr*^−/−^ +Mif blocking Ab	Experimental angioplasty	Reduced neointimal size	Chen et al. (2004)
		Reduced leukocyte recruitment	
		Reduced cell proliferation in media and neointima	
		Increased apoptosis in media and neointima	
*Apoe*^−/−^ +Mif blocking Ab	Wire injury	No significant effect on neointimal size	Schober et al. (2004)
		Reduced macrophage content	
		Increased SMC and collagen content	
**HUMAN EPIDEMIOLOGICAL STUDIES**			
*MIF* SNP rs755622 (-173 CC genotype) risk factor for CHD and diverse inflammatory diseases			Donn et al. (2001), Herder et al. (2008)
*Although not confirmed by all studies*			Palomino-Morales et al. (2010)
MIF-173 CC genotype more frequent in Turkish children with cardiomyopathy			Col-Araz et al. (2012)
MIF SNP rs1007888 (GG genotype) associated with enhanced MI risk in female Czech patients			Tereshchenko et al. (2009)
Enhanced MIF plasma levels predictive for enhanced heart failure in CHD patients with impaired glucose tolerance or type 2 diabetes mellitus			Makino et al. (2010)
Increased MIF plasma levels in patients with ACS, associated with inflammatory marker expression (CRP, IL6)			Muller et al. (2012)
**INFLAMMATORY/CARDIOVASCULAR EFFECTS OF MIF**			
*Monocytes/macrophages*			
Enhances direct monocyte recruitment and arrest through CXCR2			Bernhagen et al. (2007)
Enhances CCL2-induced monocyte recruitment			Fan et al. (2011)
Enhances oxLDL uptake and degradation			Atsumi et al. (2000)
Induces inflammatory mediators		TNFα, IL1β, IL6, IL8	Bernhagen et al. (1994), Calandra et al. (1994, 1995), Lan et al. (1997)
		NO, iNOS	Bernhagen et al. (1994), Lan et al. (1997)
Enhances expression of chemokines and adhesion molecules			Amin et al. (2006), Takahashi et al. (2009)
Interferes with p53-mediated apoptosis			Mitchell et al. (2002)
*T-cells*			
Enhances direct T-cell recruitment and arrest through CXCR2			Bernhagen et al. (2007)
*SMCs*			
*Mif*^−/−^ SMCs: reduced cathepsin expression, reduced elastin/collagen degradation capacity			Pan et al. (2004)
Inhibits long-term PDGF-BB-induced SMC migration, despite short-term stimulatory effect			Schrans-Stassen et al. (2005)
Drives SMC proliferation in some studies (but not all)			Chen et al. (2004), Schrans-Stassen et al. (2005)
*ECs*			
Enhances the (cytokine-induced) expression of chemokines and adhesion molecules			Lin et al. (2000), Gregory et al. (2006, 2009), Cheng et al. (2010)
*Other*			
Colocalizes with MMP1/9 in human vulnerable plaques			Kong et al. (2005a,b)
Pro-angiogenic			Chesney et al. (1999), Ogawa et al. (2000); Amin et al. (2003)

*Apoe, apolipoprotein E; Ldlr, low density lipoprotein receptor; ECs, endothelial cells; SMCs, smooth muscle cells; Ab, antibody; TNF, tumor necrosis factor; IL, interleukin; ICAM, intercellular adhesion molecule; MMP, matrix metalloproteinase; CEBP, CCAAT/enhancer binding protein; SNP, single nucleotide polymorphism; CHD, coronary heart disease; MI, myocardial infarction; ACS, acute coronary syndrome; CRP, C-reactive protein; oxLDL, oxidized low density lipoprotein; NO, nitrix oxide; iNOS, inducible nitric oxide synthase; PDGF, platelet-derived growth factor*.

Of note, functional animal studies confirmed an atheroprogressive role of MIF, showing a reduced lesion size and inflammatory profile in *Mif*-deficient mice, or after treatment with a Mif blocking antibody (Table 5) (Pan et al., 2004; Burger-Kentischer et al., 2006; Bernhagen et al., 2007; Verschuren et al., 2009). Remarkably, Mif blockade even induced a regression of established atherosclerotic lesions (Bernhagen et al., 2007). Similarly, Mif neutralization reduced injury-induced restenosis, in which Mif expression is initially upregulated in SMCs, and in ECs and foam cells in a later stage (Chen et al., 2004; Schober et al., 2004).

**Figure 3:**
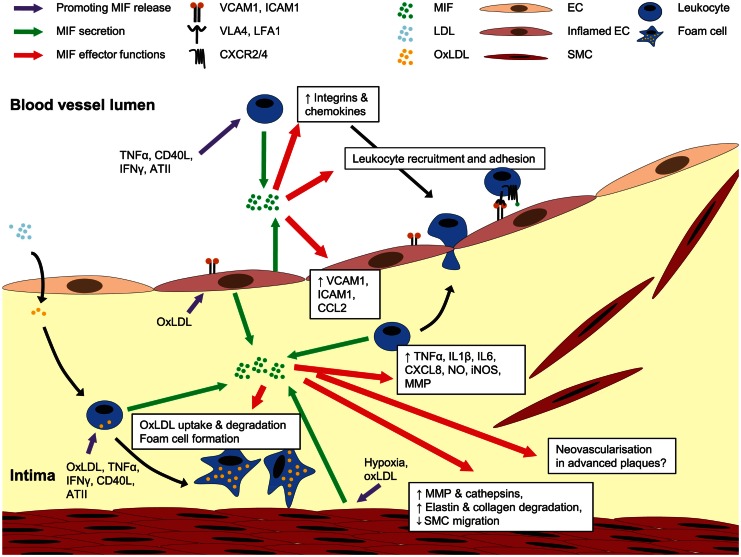
**Role of MIF in atherogenesis**. MIF is secreted upon atherogenic stimulation of ECs, SMCs, and leukocytes. Once released, MIF activates leukocyte integrins, upregulates the expression of adhesion molecules and other chemokines, together mediating leukocyte recruitment and arrest on the endothelium. MIF is also implicated in pro-inflammatory cytokine expression, transdifferrentiation of macrophages to foam cells, MMP and cathepsin induction in SMCs, and regulation of SMC proliferation and migration. A potential role for MIF in neovascularization in advanced plaques remains to be investigated. For more details, see text.

These atheroprogressive effects of MIF can be linked with MIF’s potential to trigger the expression of inflammatory mediators and mediate leukocyte recruitment and arrest directly or through the induction of adhesion molecules and chemokines in ECs and monocytes/macrophages (Figure 3) (Bernhagen et al., 1994; Calandra et al., 1994, 1995; Lan et al., 1997; Lin et al., 2000; Amin et al., 2006; Gregory et al., 2006, 2009; Takahashi et al., 2009; Cheng et al., 2010). Furthermore, the ability of MIF to stimulate oxidized LDL (oxLDL) uptake by macrophages (Atsumi et al., 2000), and its association with protease expression and a reduced PDGF-BB-induced SMC migration (Pan et al., 2004; Schrans-Stassen et al., 2005; Verschuren et al., 2005) may further contribute to MIF’s plaque destabilizing properties in hyperlipidemia-induced atherogenesis. In the context of injury-induced neointima formation, interference with MIF’s anti-apoptotic effect could underlie the enhanced apoptosis in conditions of Mif antibody treatment (Mitchell et al., 2002; Chen et al., 2004), whereas a report on MIF driving SMC proliferation could be linked to a decreased medial cell proliferation under conditions of MIF blockade (Chen et al., 2004).

Importantly, human epidemiological studies support a pro-atherogenic role of MIF (Table 5). These studies showed a single nucleotide polymorphism (SNP) in the *MIF* promotor (*MIF*-173 CC genotype) to be a risk factor for coronary heart disease (CHD) (Herder et al., 2008) and to be more frequent in Turkish children with cardiomyopathy (Col-Araz et al., 2012). SNP rs1007888 (GG genotype) was associated with enhanced risk of myocardial infarction in female Czech patients (Tereshchenko et al., 2009). Furthermore, increased plasma levels of MIF were identified as a risk factor for increased heart failure in CHD patients with impaired glucose tolerance or type 2 diabetes mellitus (Makino et al., 2010), and were associated with inflammatory marker expression in patients with acute coronary syndrome (ACS) (Muller et al., 2012).

In conclusion, multiple animal and human studies support MIF’s pro-atherosclerotic and pro-inflammatory role, and reveal MIF as an interesting target for drug development. The list of MIF inhibitors is steadily growing and includes small molecular weight or peptide drugs targeting mostly MIF’s catalytic pocket or MIF trimerization (Garai and Lorand, 2009; Ouertatani-Sakouhi et al., 2010). An interesting therapeutic strategy would be to interfere with the CLF functions of MIF by blocking the interaction of MIF with its receptors. CXCR2 and CXCR4 inhibitors have recently been discussed in more detail (Liang, 2008; Stadtmann and Zarbock, 2012). However, an attractive alternative is a direct targeting of MIF instead of its receptors or devising strategies that would specifically target the MIF/CXCR interface but not other CXCR2- or CXCR4-mediated signaling effects, i.e., as stimulated by the cognate ligands CXCL8 or CXCL12, respectively. In the context of atherosclerosis, interference with MIF binding to both CXCR2 and CXCR4 by using a MIF blocking antibody interfered with MIF’s pro-atherosclerotic functions (Bernhagen et al., 2007), while it would leave the protective homeostatic functions of the CXCL12-CXCR4 axis preserved (Koenen and Weber, 2010). Specific targeting of binding motifs in MIF, e.g., the pseudo-(E)LR and the N-like loop motifs critical for the MIF-CXCR2 interaction, provides an interesting strategy, but MIF motifs crucial for MIF-CXCR4 binding still remain to be identified. Finally, it is important to keep in mind that MIF exerts pleiotropic functions, and also behaves protective in different settings. For example, the MIF-CD74 axis is cardioprotective after myocardial ischemia/reperfusion injury (Miller et al., 2008; Qi et al., 2009; Luedike et al., 2012), and also exerts an important antifibrotic effect in experimental liver fibrosis (Heinrichs et al., 2011). Also, MIF polymorphisms associated with higher MIF expression were found to have a beneficial effect in community-acquired pneumonia (Yende et al., 2009). Therefore, possible negative side effects should always be carefully monitored for each new MIF inhibitor.

## Pericytes Coordinate Interstitial Leukocyte Migration through MIF

MIF plays a pivotal role in leukocyte chemotaxis in the blood and other body fluids, in the arrest of leukocytes on the endothelium and their transmigration into the sub-endothelial space (Gregory et al., 2006; Bernhagen et al., 2007; Cheng et al., 2010; Santos et al., 2011). Intriguingly, it was recently shown that MIF also plays a role in directing extravasated leukocytes in the peri-endothelial compartment to NG2^+^ pericyte-rich regions along arterioles and capillaries (Stark et al., 2013) (Box 4). Stark et al. (2013) identified NG2^+^ pericytes as the main source of MIF in the perivascular compartment of microvessels and demonstrated that stimulation of these cells with pro-inflammatory stimuli such as TNF, LPS, or damage-associated molecular patterns (DAMPs) released MIF and immobilized it on the cell surface of pericytes, probably through binding to CD74 and/or CXCR4. Furthermore, the release of MIF, CCL2, and CXCL8, together with the expression of ICAM1 on the pericyte surface mediated monocyte and neutrophil chemotaxis in an LFA1- and MAC1-dependent manner. As leukocyte extravasation occurs only in the postcapillary venules, which are covered by NG2^−^ pericytes, but not in arterioles and capillaries containing NG2^+^ pericytes, neutrophil and macrophage interaction with NG2^+^ pericytes can only occur after successful interstitial migration from their entry point in postcapillary venules toward capillary and arteriolar pericytes, providing interstitial migration routes for the extravasated leukocytes (Murfee et al., 2005; Stark et al., 2013). The importance of MIF in this leukocyte migration track in the pericyte sheath was stressed by the observation that subcutaneous injection of the MIF inhibitor ISO-1 in mice reduced the number of accumulated neutrophils around NG2^+^ pericytes in the skin microvasculature without affecting their extravasation (Stark et al., 2013). This finding, together with the capacity of MIF to induce CCL2-dependent leukocyte extravasation in postcapillary venules (Gregory et al., 2006), supports the concept, that the gradual interplay of MIF with different cell types, other chemokines, or inflammatory mediators is important for successful MIF-mediated leukocyte direction from the blood vessel lumen to the site of inflammation.

Box 4 **Pericytes.**

Pericytes are also known as mural or rouget cells. They are essential components of microvessels, in which they are closely associated with the microvessel ECs, enveloped in a common basement membrane. Pericytes adhere to matrix proteins like fibrinogen, laminin, and collagen of the basement membrane through integrins. Due to their morphological and phenotypical heterogeneity, it is hard to distinguish them from other peri-endothelial cells, causing them to be often mixed up with vascular SMCs or mesenchymal cells. Also, no specific marker has been found yet. For example, pericytes covering postcapillary venules are NG2^−^ αSMA^+^, whereas pericytes on arterioles and capillaries are NG2^+^. Some commonly used markers are α-smooth muscle actin (αSMA), alanyl(membrane)aminopeptidase (CD13), chondroitin sulfate proteoglycan 4 (NG2), melanoma cell adhesion molecule (CD146), platelet-derived growth factor receptor (PDGFR)-β, and desmin.

## Concluding Remarks

MIF has been recognized as an important CLF chemokine mediating leukocyte recruitment and arrest in the context of many inflammatory diseases, in particular atherosclerosis. The receptors CD74, CXCR2, and CXCR4 have been identified to bind MIF and to mediate MIF-triggered arrest functions, but the molecular mechanisms underlying MIF-mediated receptor signaling toward different cellular functions still need further refinement. For example, the molecular sequelae of MIF-triggered events leading to integrin activation on leukocytes is still largely unexplored. In addition, the composition, balance, and differential functionality of different MIF receptor complexes needs further investigation. Identification and refinement of the critical receptor binding sites of MIF could stimulate the search for drugs that specifically interfere with MIF binding to only one or a certain selection of MIF receptors, with the aim to selectively interfere with only a subset of MIF’s pleiotropic functions.

## Conflict of Interest Statement

The authors declare that the research was conducted in the absence of any commercial or financial relationships that could be construed as a potential conflict of interest.

## Footnotes

^1^Throughout the manuscript, the letter format of all gene and protein notations was chosen to conform with internationally agreed gene/protein nomenclature guidelines: all letters of human genes/proteins are in uppercase, whereas for mouse genes/proteins, only the first letter is in uppercase and the remaining letters are in lowercase. Gene names are in italics.

^2^MIF tautomerase activity requires trimerization.

^3^In the paper by Fingerle-Rowson et al. (2009), MIF sequence numbering refers to the processed MIF protein after Met1 removal. Thus, the protein sequence of MIF in that paper starts with Pro1 rather than Pro2, the latter referring to the cDNA sequence. In the current article, we prefer to refer to this proline residue as Pro2, i.e., applying the numbering according to the cDNA sequence to be consistent with the sequence numbering of the mutants above.
